# Speech Recognition via fNIRS Based Brain Signals

**DOI:** 10.3389/fnins.2018.00695

**Published:** 2018-10-09

**Authors:** Yichuan Liu, Hasan Ayaz

**Affiliations:** ^1^School of Biomedical Engineering, Drexel University, Science and Health Systems, Philadelphia, PA, United States; ^2^Cognitive Neuroengineering and Quantitative Experimental Research (CONQUER) Collaborative, Drexel University, Philadelphia, PA, United States; ^3^Department of Family and Community Health, University of Pennsylvania, Philadelphia, PA, United States; ^4^The Division of General Pediatrics, Children's Hospital of Philadelphia, Philadelphia, PA, United States

**Keywords:** BCI, fNIRS, prefrontal cortex (PFC), parietal lobe, speech perception, decoding

## Abstract

In this paper, we present the first evidence that perceived speech can be identified from the listeners' brain signals measured via functional-near infrared spectroscopy (fNIRS)—a non-invasive, portable, and wearable neuroimaging technique suitable for ecologically valid settings. In this study, participants listened audio clips containing English stories while prefrontal and parietal cortices were monitored with fNIRS. Machine learning was applied to train predictive models using fNIRS data from a subject pool to predict which part of a story was listened by a new subject not in the pool based on the brain's hemodynamic response as measured by fNIRS. fNIRS signals can vary considerably from subject to subject due to the different head size, head shape, and spatial locations of brain functional regions. To overcome this difficulty, a generalized canonical correlation analysis (GCCA) was adopted to extract latent variables that are shared among the listeners before applying principal component analysis (PCA) for dimension reduction and applying logistic regression for classification. A 74.7% average accuracy has been achieved for differentiating between two 50 s. long story segments and a 43.6% average accuracy has been achieved for differentiating four 25 s. long story segments. These results suggest the potential of an fNIRS based-approach for building a speech decoding brain-computer-interface for developing a new type of neural prosthetic system.

## Introduction

The decoding of speech from brain signals has attracted the attention of researchers in recent years (Chakrabarti et al., 2015; AlSaleh et al., 2016; Herff and Schultz, 2016). A device that can directly translate brain signals into texts that describe a person's thoughts may help people with disabilities and verbal communication deficits and enable a new communication channel with the outside world. Such brain-computer interfacing device may also help healthy people to directly interact with a machine without the need of using muscles and potentially expand the interaction bandwidth.

Most of the previous studies focused on phoneme-based decoding and adopted invasive or partially invasive technology that requires the implant of sensors during neurosurgery. For example, Brumberg et al. investigated the classification of intended phoneme production based on intracortical microelectrode recordings (Brumberg et al., 2011). Herff et al. decoded words from continuously spoken speech from intracranial electrocorticographic (ECoG) signals recorded from epileptic patients based on the classification of phonemes (Herff et al., 2015). A 75% classification accuracy has been achieved for a dictionary of 10 words and a 40% accuracy for a dictionary of 100 words. Martin et al. investigated the classification of individual words from a pair of imagined word using ECoG and a 58% binary classification accuracy has been achieved (Martin et al., 2016). The authors also showed that the binary classification of listened and overt spoken words is much better, which achieved 89% and 86% accuracy, respectively. We refer to Chakrabarti et al. (2015) and Herff and Schultz (2016) for a more comprehensive review of the state of art in the field.

Despite of the promising results achieved, invasive technology requires the implantation of sensors which limits their applications, especially for the healthy populations. In the field of non-invasive brain-computer interface, studies mainly adopted electroencephalography (EEG) and functional magnetic resonance imaging (fMRI) for the classification of speech related task conditions. For example, O'Sullivan et al. played two audio stories of 60 s long simultaneously to the subjects who were instructed to attend to one of the stories. They were able the identify which story the subjects were attended to with 89% accuracy (O'Sullivan et al., 2015). Yoshimura et al. investigated the 3-class classification of two imagined phonemes and no-imagery control and a 59% accuracy has been achieved adopting fMRI prior for current source estimation (Yoshimura et al., 2016). Vodrahalli et al. investigated the classification of movie scene from fMRI response (Vodrahalli et al., 2017). A shared response model (SRM) has been used for dimension reduction and a classification accuracy of 72% has been achieved over 4% chance level. A recent fMRI study investigated a similar task of identifying musical pieces (Hoefle et al., 2018). A multiple regression has been adopted to predict features of musical pieces for the task of differentiating between new pieces not used for training the predictive model.

In this study, we adopted fNIRS for the classification of listened stories. This approach is motivated from our recent fNIRS study (Liu et al., 2017c) and a number of other fMRI studies (Stephens et al., 2010; Lerner et al., 2011; Hasson et al., 2012) which show that listeners' brain mirror each other whenever they are listening to the same story and the listeners' brain mirror the speaker's brain with a delay. We also inspired from fNIRS BCI studies which revealed the potential of fNIRS in classifying mental conditions (Ayaz et al., 2009; Power et al., 2010, 2012; Fazli et al., 2012a,b; Khan et al., 2014; Liu et al., 2017b).

fNIRS is an optical based neuroimaging technique for measuring the cortical concentration changes in the oxygenated (oxy-Hb) and deoxygenated (deoxy-Hb) hemoglobin. It is portable, wearable (Piper et al., 2014; Mckendrick et al., 2015), non-invasive and can even be battery-operated and wireless, and hence particularly suitable for brain-computer interfacing in everyday settings (Ayaz et al., 2011, 2013; Liu et al., 2017a,b). Several studies in the literature adopted fNIRS to investigate the classification of neural signals during listening comprehension, speech production or related topics. Power et al. (2010) investigated the classification of music and mental arithmetic conditions and a 77% accuracy has been achieved (Power et al., 2010). The same group in 2012 investigated the three-class classification problem of differentiating mental arithmetic, mental singing and no-control state and a 56.2% accuracy has been achieved (Power et al., 2012). Telkemeyer et al. (2011) investigated the acoustic processing of non-linguistic sounds in infants combining EEG and fNIRS (Telkemeyer et al., 2011). Herff et al. (2012) adopted fNIRS for differentiating between speech and not speaking conditions (Herff et al., 2012). They achieved 71% and 61% for classifying overt speech/not speaking and silent speech/not speaking, respectively. Moghimi et al. (2012) investigated the classification of music excerpts with different emotional content using only fNIRS. They were able to differentiate excerpts with positive and negative emotions with 72% accuracy (Moghimi et al., 2012). Putze et al. (2014) combined EEG and fNIRS for differentiating visual and auditory perception processes from each other and achieved 98% accuracy (Putze et al., 2014).

To study fNIRS-based speech recognition, we used the data from our previous study which included fNIRS recordings while participants (*N* = 19) were listening to English stories (Liu et al., 2017c). We divided the fNIRS signals into several segments (each corresponding to a part of a story) and machine learning was applied for identifying which part of the stories a participant was listening to using only fNIRS signal.

A major obstacle in the classification of fNIRS signal is the individual variations caused by the different size and shape of the head/brain across the subjects. For some subjects, their head shape resulted in channels being rejected due to bad contact. Conventionally, this problem can be alleviated by acquiring additional information such as the 3D coordinates of the sensors and results from a structural magnetic resonance imaging scan. This information can be used to estimate the exact location of the brain a sensor was measuring from and transform all data to a standard brain space (Tsuzuki and Dan, 2014). However, the conventional approach can be time consuming, costly and it doesn't take into account the individual differences in brain activation regions which could also deteriorate the accuracy of fNIRS signal classification. As a solution to this problem, we applied spatial filters for extracting latent variables that have minimum between-subject variations. Spatial filters find linear combination of the optodes (i.e., fNIRS sensors) to linearly transform the raw data. We consider two spatial filtering techniques: GCCA (Shen et al., 2014) and SRM (Chen et al., 2015). Each of these techniques finds a set of subject-specific spatial filters to extract latent variables. GCCA extracts the 1st latent variable that maximizes the between-subject correlations in the signal time course. It then extracts the 2nd latent variable to maximize the between-subject correlations subject to the constrain that it is uncorrelated with the 1st latent variable. This procedure is repeated until no more latent variable could be found. For SRM, it finds spatial filters which minimize the sum of squared error between extracted latent variables and the estimated component time courses that are shared among all subjects.

## Methods

### Participants

Nineteen participants from our previous study (Liu et al., 2017c) who have completed the listening comprehension of both story E1 and E2 were used for the analysis in this study.

### Signal acquisition

**Figure 1:**
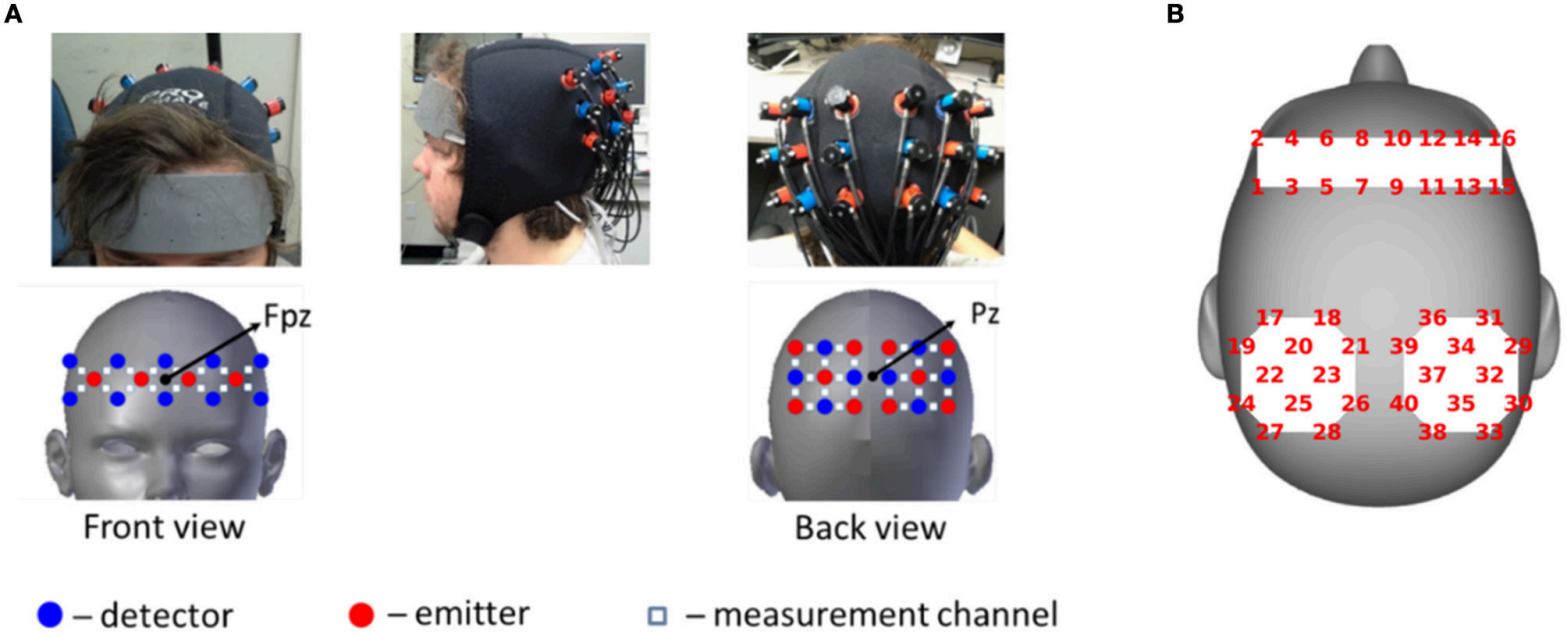
**(A)** fNIRS acquisition setup. Red circles indicate emitters; blue circles indicate detectors; White squares indicate measurement channels between emitters and detectors. **(B)** Schematic representation of the same optode locations on head surface which is used to show results. Written informed consent was obtained from the participant and adapted from Liu et al. (2017c).

Two continuous wave optical brain imaging devices were used simultaneously on each participant to record brain activity from prefrontal cortex (PFC) and parietal cortex (PL) using 40 measurement locations (optodes) (Figure 1). Anterior prefrontal cortex was recorded in 2 Hz by a 16-optode continuous wave fNIRS system (fNIR Imager Model 1100; fNIR Devices, LLC) first described by Chance et al. (1998) and developed in our lab at Drexel University (Izzetoglu et al., 2005; Ayaz et al., 2011). Parietal cortex was recorded in 10 Hz using a 24-optode continuous wave Hitachi fNIRS system (ETG 4000; Hitachi Medical Systems). Please refer to Liu et al. (2017c) for a detailed description of the signal acquisition.

### Signal processing

Raw light intensities were converted into concentration changes in oxygenated-hemoglobin (HbO) and deoxygenated-hemoglobin (HbR) concentrations using the modified Beer-Lambert law (Cope and Delpy, 1988). The raw signal and hemoglobin concentration changes were inspected both visually and also using the automated SMAR algorithm (Ayaz et al., 2010), which uses a coefficient-of-variation based approach to assess signal quality, reject problematic channels with bad contact or saturated raw light intensity. Two optodes, 1 and 15, were over the hairline for most participants and hence were rejected from the study. One more optode (optode 37) was rejected from the study because it has been rejected in more than 40% of the subjects. The HbO and HbR signals were band-pass filtered from 0.01 to 0.1 Hz using a 4-th order zero-phase infinite impulse response (IIR) filter for reducing artifacts from physiological signals (Ayaz et al., 2011). The cut-off frequencies reflect common settings addressing global drifts (<0.01 Hz) and systemic interferences such as Mayer wave (~0.1 Hz), respiration rate (>0.2 Hz) and cardiac cycles (>0.5 Hz). We then downsampled the signals to 1 Hz. We rejected the first 30 s of each story because they may be affected by transient global increases or decreases in response amplitude caused by the start of listening comprehension. For each story, the signal time courses were divided into segments of 100 s duration. A total of 9 segments were extracted from the two stories.

### Feature extraction

fNIRS signals vary considerably from subject to subject due to the different head size, head shape, and spatial locations of brain functional regions. For reducing the between-subject variations, two spatial filtering approaches were considered: the shared response model (SRM) and generalized canonical correlation analysis (GCCA).

#### Shared response model

The SRM was proposed by Chen et al. (2015) for identifying the shared brain responses among subjects by estimating subject-specific spatial filters. More specifically, the spatial filters ***W***_***i***_ for subject *i* were estimated as below:

(1) $$\begin{array}{l} \min_{wi},s\sum_{i}\|W_{i}^{T}X_{i}-S{\|}_{F}^{2}\\ \;s.t.W_{i}^{T}W_{i}=I_{k} \end{array}$$

where $X_{i}\in {\mathbb{R}}^{v_{i}\times t}\left(i=1,\ldots ,N\right)$ is the fNIRS signals of subject *i* with *v*_*i*_ channels and *t* time points. In this study $\underset{i}{\max}(v_{i})=37(optodes)\times 2\left(HbO/HbR\right)=74$. For a subject, some optodes were rejected due to over the hairline, ambient light leakage or severe motion artifact contamination. $W_{i}\in {\mathbb{R}}^{v_{i}\times k}$ is the spatial filters of subject *i* and the parameter *k* represents the number of spatial filters to be estimated. Parameter *k* needs to be selected by the experimenter. And ***S*** ∈ ℝ^*k*×*t*^ is the corresponding time series of responses shared by all participants. For each subject, ${\tilde{X}}_{i}=W_{i}^{T}X_{i}$ is then used as feature for classification.

#### Generalized canonical correlation analysis

GCCA estimates subject-specific spatial filters for extracting orthogonal components that are maximally correlated among the subjects. We denote $X_{i}\in {\mathbb{R}}^{v_{i}\times t}\left(i=1,\ldots ,N\right)$ the fNIRS signals of subject *i* with *v*_*i*_ channels and *t* time points and $W_{i}\in {\mathbb{R}}^{v_{i}\times k}\left(i=1,\ldots ,N\right)$ the spatial filters for subject *i*. GCCA maximizes the following:

(2) $$\begin{array}{l} \phi \left(W\right)=tr\left(W^{T}XX^{T}W\right)\\ s.t.W^{T}DW=I_{t} \end{array}$$

where $X={\left[X_{1}^{T},\ldots ,X_{N}^{T}\right]}^{T}\in {\mathbb{R}}^{v\times t}$ (*v* = *v*_1_ + … + *v*_*N*_), $W={\left[W_{1}^{T},\ldots ,W_{N}^{T}\right]}^{T}\in {\mathbb{R}}^{v\times k}$ and $\text{D}=\left(\begin{array}{ccc} X_{1}X_{1}^{T} & \cdots & 0\\ \vdots & \ddots & \vdots \\ 0 & \cdots & X_{N}X_{N}^{T} \end{array}\right)$is a block diagonal matrix. This could be achieved by solving the following generalized eigenvalue problem:

$$\begin{array}{l} XX^{T}w=\lambda Dw \end{array}$$

After the spatial filters were estimated, the latent variables ${\tilde{X}}_{i}=W_{i}^{T}X_{i}$ is used as features for classification.

### Inter-subject correlation

For gaining an intuitive understanding of the relative importance of the fNIRS channels in characterizing the story segments, we calculated the inter-subject correlation for each fNIRS channel *j* as follows:

$$\begin{array}{l} r^{j}=\frac{1}{19}\overset{19}{\underset{i=1}{\sum }}\rho \left(x_{i}^{j},{\bar{x}}_{i}^{j}\right) \end{array}$$

where ρ(·) denotes Pearson's correlation, $x_{i}^{j}$ is the fNIRS time course of subject *i*, ${\bar{x}}_{i}^{j}$ is the average time course of all other subjects except subject *i*. The inter-subject correlation reflects the consistency of the signal cross different subjects. To test the significance of the inter-subject correlation, a phase-scrambling random permutation procedure as used in (Lerner et al., 2011) was adopted. More specifically, the Fourier transform was applied on a fNIRS time course, the phase of the frequency components was randomized and the inverse Fourier transform was applied to obtain a phase-scrambled time course. This procedure was repeated 1,000 times for estimating the null distributions of the inter-subject correlations. In our study, there are 34 (optodes) × 2 (HbO/HbR) = 74 fNIRS channels. To correct for multiple comparison, the maximum statistic approach was applied (Nichols and Holmes, 2002), i.e., in each of the 1,000 iterations, the maximum inter-subject correlation values among the 74 phase-scrambled fNIRS time courses were calculated (*r*_*max*_ = max_*j* = 1…74_*r*_*j*_) for estimating the null distribution of *r*_max_. The null hypothesis of a fNIRS channel is rejected if its inter-subject correlation is higher than 95% of the samples in the null distribution of *r*_max_.

### Classification and performance evaluation

For classification, the fNIRS time courses of the signal segments were used as feature, a principal components analysis was adopted for dimensional reduction and the logistic regression was adopted for classification. A leave-one-segment-out cross-validation was performed to apply spatial filtering for reducing between-subject variations and a 10-fold cross-validation was performed to evaluate story segments classification. We denote ${\text{X}}_{i}^{p}\left(i=1,\ldots ,N;p=1,\ldots ,9\right)$ the fNIRS time courses of subject *i* and story segment *p*. The performance evaluation procedure is as follows:

For story segment *p* = 1, …, 9:

- Spatial filter training:
- All story segments except segment *p* $\left\{{\text{X}}_{i}^{j},j\neq p,\;i=1,\ldots ,N\right\}$ were used as training set for estimating *K* spatial filters for each subject (${\text{W}}_{\text{p}}={\left[{\text{W}}_{1\text{p}}^{\text{T}},\ldots ,{\text{W}}_{\text{N}\text{p}}^{T}\right]}^{T}\in {\mathbb{R}}^{v\times K}$) adopting SRM or GCCA. Before applying spatial filtering, the fNIRS optodes were normalized to have a mean of zeros and standard deviation of ones. The 30 s. long data immediately before and after the testing segments were rejected from training data.
- Spatial filter testing:
- The spatial filters estimated in training were applied to the story segment *p* (the testing set) to extract spatial components ${\tilde{X}}_{i}^{p}$.
- Classification:
- The estimated ${\tilde{\text{X}}}_{i}^{p}$ (or ${\text{X}}_{\text{i}}^{\text{p}}$ if spatial filtering is not applied**)** were divided into four 25 s sub-segments (four-class classification problem) or two 50 s sub-segments (binary classification problem) for investigating story segments classification. For the four-class classification problem, there were 4 (classes) × 19 (subjects) = 76 samples and each sample included *k* (latent variables) × 25 (time points) = 25*k* features. For the two-class classification problem, there were 2 (classes) × 19 (subjects) = 38 samples and each sample included *k* (latent variables) × 50 (time points) = 50*k* features. The 10-fold cross-validation was then applied to the 76 (four-class problem) or 38 (two-class problem) samples for estimating the classification accuracy. More specifically, we randomly divided the subjects into 10 groups. Using the data from 9 groups as training subjects, we first applied PCA to reduce the dimensionality of the data. The largest principal components that explained 99.9% of the variance of the data were extracted. A logistic regression analysis was performed using the principal components as features. The classification accuracy of the group that has been left out was then estimated applying the PCA and classifier coefficients learned from training data. The classification accuracy using story segment *p* as testing segment and subject *i* as one of the testing subject is denoted as $acc_{i}^{p}$.

**Figure 2:**
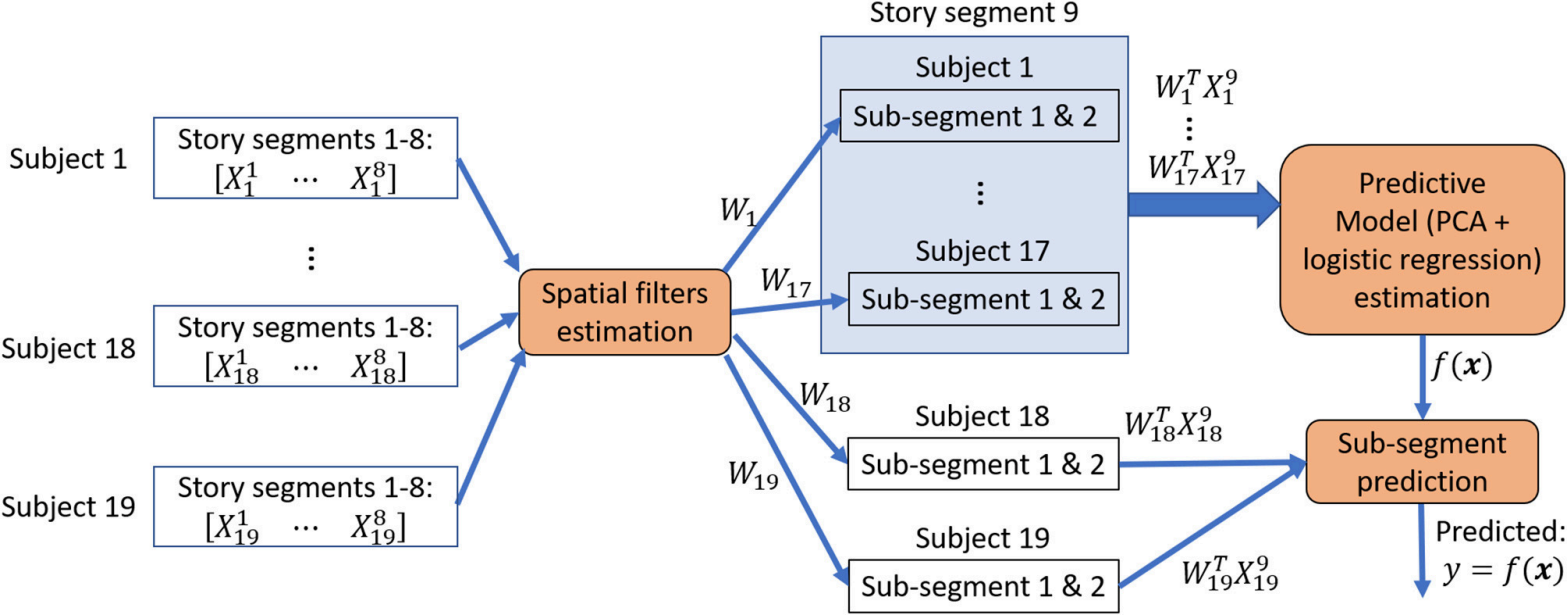
Story sub-segments classification procedure. Spatial filters were estimated from 8 story segments to minimize between-subject variations and then applied to the 9th segment that has been left out. The 30 s. of data immediately before and after the 9th segment was rejected from training data. This procedure is repeated until all segment has been left out exactly once for a leave-one-segment-out cross-validation. During each iteration of the cross-validation, the spatially filtered segment that has been left out were further divided into 2 or 4 sub-segments, forming a 2-class or 4-class classification problem. The sub-segments from 18 or 19 subjects were used to estimate a predictive model and its classification performance is evaluated on the one or two subjects that has been left out for performing an inner 10-fold cross-validation.

For each subject *i*, we compared the average classification accuracy $acc_{i}=\frac{1}{9}\overset{9}{\underset{p=1}{\sum }}acc_{i}^{p}$achieved with SRM-estimated spatial filter, with GCCA-estimated spatial filter and without applying any spatial filter. Figure 2 illustrates the procedure for estimating story segments classification accuracies.

## Results

### Inter-subject correlation

**Figure 3:**
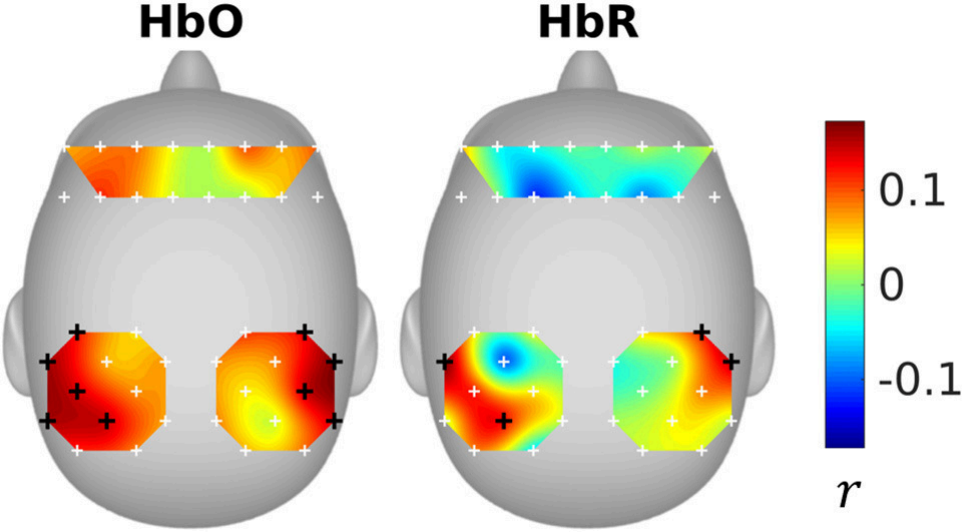
Inter-subject correlation. Black “+” sign represents significant channels.

The inter-subject correlations were shown in Figure 3. It can be seen that the subjects were significantly correlated in the parietal areas. The significant optodes cover parts of supramarginal gyrus, angular gyrus, superior parietal gyrus, and postcentral gyrus (please refer to Figure 8 and Table S1 of Liu et al., 2017c, for the fNIRS optode locations).

### SRM estimated spatial filters

**Figure 4:**
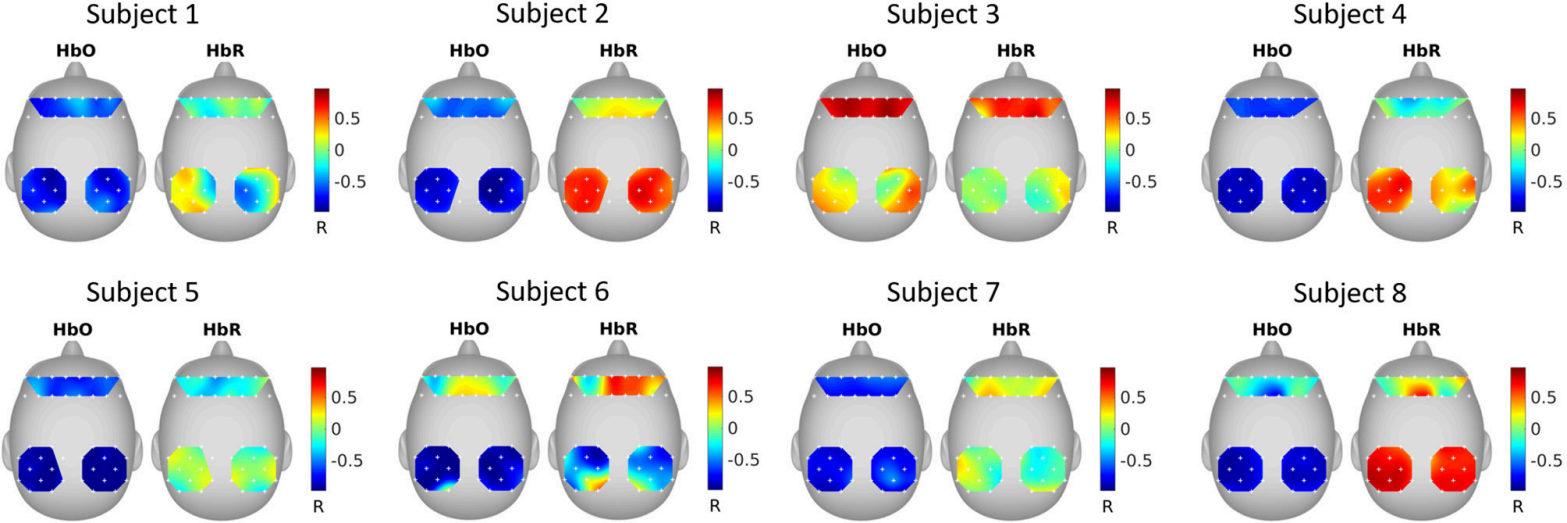
SRM estimated spatial filters. The spatial filters for each subject were estimated with SRM *k* = 1 spatial filter. The topographical maps show the Pearson's correlation between the extracted latent variable and the fNIRS channels.

Figure 4 shows the correlation between SRM extracted latent variable (*k* = 1 *spatial filter*) and fNIRS channels. It can be seen that the latent variable is mostly negatively correlated with HbO and positively correlated with HbR.

### GCCA estimated spatial filters

**Figure 5:**
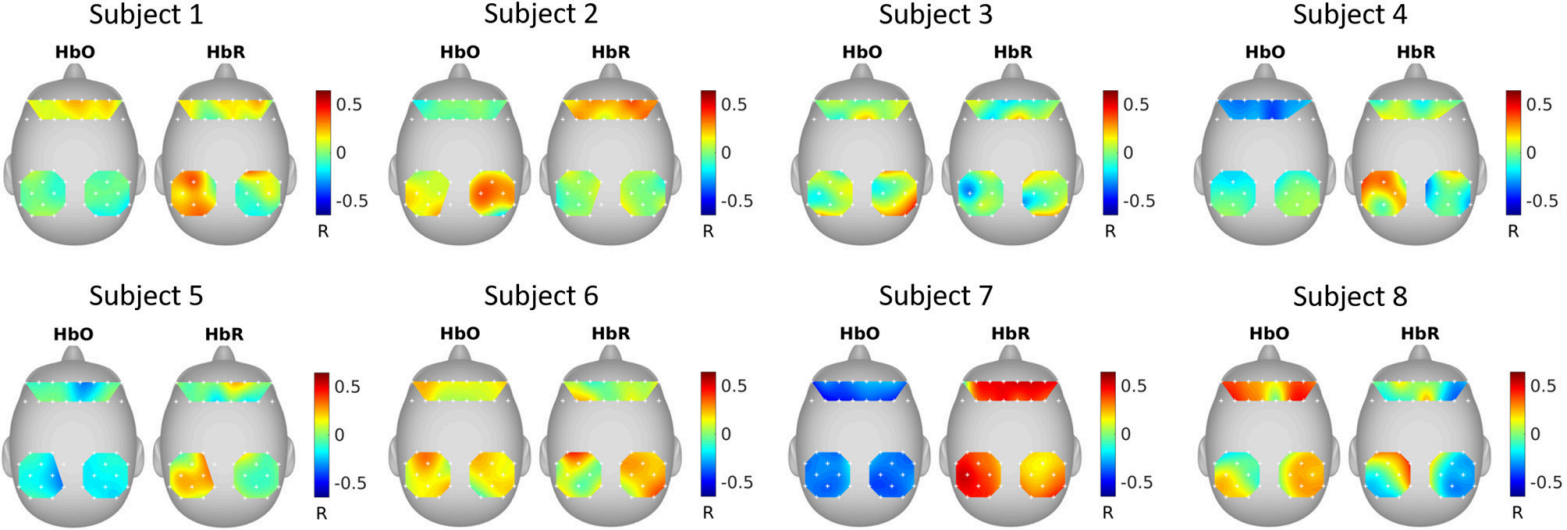
GCCA estimated spatial filters. The topographical maps show the Pearson's correlation between the extracted latent variable that was most correlated among subjects and the fNIRS channels.

Figure 5 shows the correlation between GCCA extracted latent variable (the variable that is most correlated among subjects) and fNIRS channels. It can be seen that the correlation pattern varies for different subjects.

### Classification

**Figure 6:**
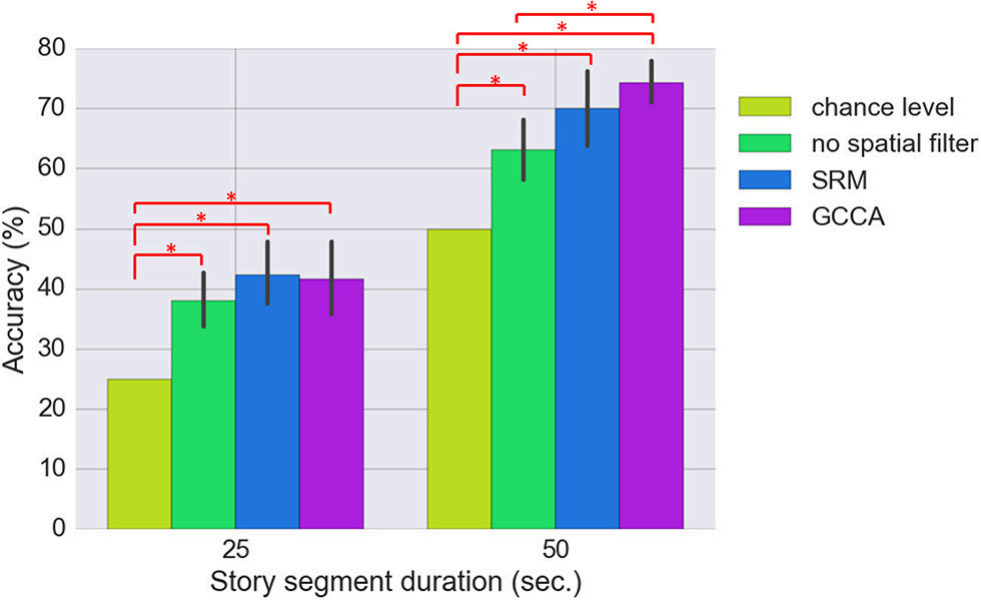
Comparing the classification accuracy using SRM (*K* = 74 latent variables), GCCA (*K* = 74 latent variables), and without spatial filter. The results for two classification problems are shown: 4-class (25 s. sub-segment duration) and binary (50 s. sub-segment duration). The error bar stands for bootstrapped 95% confidence interval. Methods with statistically significant differences (FDR *q* < 0.05) are marked by red bars and asterisks.

Figure 6 shows the classification results for the three approaches (SRM, GCCA or no spatial filtering) with different story segment duration. We first estimated using all 74 spatial components for classification [there are 37 (optodes) × 2 [HbO/HbR] = 74 channels]. All three approaches achieved significantly better than chance level accuracy (FDR *q* < 0.05). The chance level accuracy is 50% for the binary classification and 25% for the 4-class classification problem. Without spatial filtering, 63.2 ± 11.8% and 38.0 ± 9.8% (mean ± standard deviation) accuracy have been achieved for the two-class (50 s. sub-segment) and four-class (25 s. sub-segment) problem, respectively. GCCA achieved 74.7 ± 8.5% and 43.6 ± 13.2% accuracy for the two-class problem and four-class problem, respectively. For the two-class problem, GCCA significantly outperformed the accuracy achieved without spatial filtering (FDR *q* < 0.05). SRM achieved 72.0 ± 10.5% and 43.8 ± 12.1% accuracy for the two-class and four-class problem, respectively. No other significant differences between the three approaches has been found.

**Figure 7:**
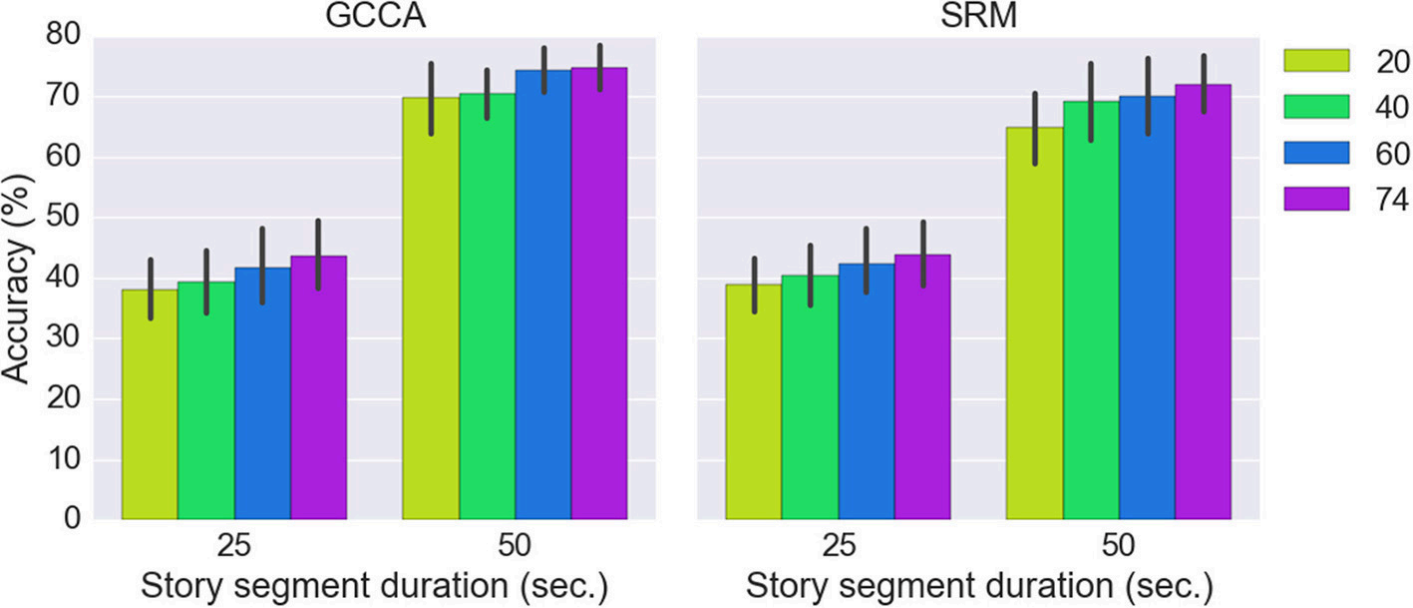
Classification accuracies of GCCA and SRM with different number of spatial components.

The effect of different number of spatial components on GCCA and SRM accuracy is shown in Figure 7. It can be seen that the highest accuracy was achieved using all 74 spatial components.

## Discussion

In this study, we applied machine learning to identify among several story segments the one that was listened by a subject based on the brain's hemodynamic response measured with fNIRS. An inter-subject correlation analysis revealed that the time courses of fNIRS were significantly correlated at parietal areas, suggesting that signal was most consistent at parietal optodes, and parietal optodes may have provided the most information for characterizing the story segments. To reduce the between-subject variations caused by inter-subject differences such as head size, head shape, and brain activation regions, spatial filters were applied to extract latent variables which are linear combinations of the fNIRS optodes. Without spatial filtering, a story segment classification accuracy of 63.2% and 38.0% have been achieved for the binary classification problem (50 s. story segment duration) and 4-class classification problem (25 s. story segment duration), respectively. After applying GCCA spatial filters, a classification accuracy of 74.7% and 43.6% have been achieved for the binary and 4-class classification problems, respectively. This is better than the results achieved without spatial filtering. Applying SMR spatial filters resulted in better classification accuracy compared to no spatial filter but it is not as effective as GCCA especially for the binary classification problem.

Although we performed the classification of fNIRS signals in response to the listening comprehension of stories, we speculate that it is also plausible to classify overt or even covert speech production based on the evidences that a listener's brain mirrors the speaker's brain with a delay (Stephens et al., 2010; Liu et al., 2017c). Further evidences can be found in an fNIRS-based speaking mode classification study (Herff et al., 2012). In the study, the binary classifications of overt speech/not speaking and silent speech/not speaking were investigated and an accuracy of 71% and 61% have been achieved, respectively.

It is worth pointing out that we only utilized fNIRS from 37 locations in the prefrontal and parietal lobe. With full head fNIRS coverage and increased optode density, we expect an even better story segment classification performance for allowing the identification of shorter speech from a larger pool of candidate speeches.

Despite the promising results, the current study is limited in the following aspects. First, the audio clips have been played to the subjects only once, i.e., the stories were novel to all subjects. How well the predictive models can perform for stories that has been repeatedly played to the subjects are still unknown. Second, we evaluated the performance of the spatial filters on story segments with a total duration of 900 s. The stories have been played to the participants on the same day within 2 h. How well the spatial filters can generalize to longer stories and for stories played on different days remain to be seen. We speculate that estimating the spatial filters using longer stories with more varieties in the story content and applying regularization techniques can help improve generalization. Finally, our accuracy still needs improvement for real-world setups and 25–50 s time course length is not suitable for most of the neuroprosthetic applications. Further development in fNIRS signal acquisition and processing is needed for improving decoding accuracy and decreasing the time course length. Incorporating information from other modalities such as EEG may also help.

In summary, we showed that it is possible to identify speech from fNIRS data with machine learning techniques. The application of spatial filters reduced the inter-subject variations in the data and help improved classification performance. The current study is a step toward a BCI that reconstructs speech contents from brain signals for helping locked-in syndrome patients or healthy individuals to augment human-computer interaction as a new type of neural prosthetic system. However, there is a long way before such BCI can be achieved. As the next step, studies could be conducted using high density fNIRS covering more areas of the brain and incorporating information from other modalities such as EEG for improving the classification accuracy of shorter speeches.

## Ethics statement

This study was carried out in accordance with the recommendations of Institutional Review Board of Drexel University. The protocol was approved by the Institutional Review Board of Drexel University. All subjects gave written informed consent in accordance with the Declaration of Helsinki.

## Author contributions

YL performed the experiment, collected the fNIRS data, analyzed the data, and prepared/wrote the manuscript. HA initiated and supervised the study, designed the experiment, analyzed the data, discussed, and interpreted the results as well as prepared/revised the manuscript.

### Conflict of interest statement

fNIR Devices, LLC manufactures the optical brain imaging instrument and licensed IP and know-how from Drexel University. HA was involved in the technology development and thus offered a minor share in the new startup firm fNIR Devices, LLC. The remaining author declares that the research was conducted in the absence of any commercial or financial relationships that could be construed as a potential conflict of interest.
